# Wild leafy vegetables: A study of their subsistence dietetic support to the inhabitants of Nanda Devi Biosphere Reserve, India

**DOI:** 10.1186/1746-4269-4-15

**Published:** 2008-05-30

**Authors:** Shalini Misra, RK Maikhuri, CP Kala, KS Rao, KG Saxena

**Affiliations:** 1 G.B. Pant Institute of Himalayan Environment and Development, Garhwal Unit, P O Box 92, Srinagar (Garhwal) 246 174, India; 2 National Medicinal Plants Board, Ministry of Health and Family Welfare, Government of India, 36-Janpath, Chandralok Building, New Delhi 110 001, India; 3 Center for Inter-disciplinary Studies of Mountain and Hill Environment (CISMHE), Academic Research Center, University of Delhi, Delhi 110 007, India; 4 School of Environmental Sciences, Jawaharlal Nehru University, New Delhi 110 067, India

## Abstract

Consumption of greens is a major source of vitamins and micro-nutrients for people using only vegetarian diets rich in carbohydrates. In remote rural settlements where vegetable cultivation is not practiced and market supplies are not organized, local inhabitants depend on indigenous vegetables, both cultivated in kitchen gardens and wild, for enriching the diversity of food. Knowledge of such foods is part of traditional knowledge which is largely transmitted through participation of individuals of households. A total of 123 households in six villages of Nanda Devi Biosphere Reserve buffer zone was surveyed using a schedule to assess the knowledge, availability and consumption pattern of wild leafy vegetables. Quantity estimations were done using regular visits with informants from 30 sample households of the six study villages during the collections. Monetization was used to see the value of wild leafy vegetables harvested during a year. The diversity of wild leafy vegetables being use by the local inhabitants is 21 species belonging to 14 genera and 11 families. This is far less than that being reported to be used by the communities from Western Ghats in India and some parts of Africa. Irrespective of social or economic status all households in the study villages had the knowledge and used wild leafy vegetables. The number of households reported to consume these wild leafy vegetables is greater than the number of households reporting to harvest them for all species except for *Diplazium esculentum *and *Phytolacca acinosa*. The availability and use period varied for the species are listed by the users. The study indicated that the knowledge is eroding due to changing social values and non participation of younger generation in collection and processing of such wild leafy vegetables.

## Background

Since time immemorial useful plants have been handled by human societies for medicinal and food purposes. While, the hunter-gatherer societies still continue to profess such lifestyles, the agricultural societies did not eliminate the use of non-cultivated resources. Today, most human plant food is based on rather limited number of crops (12 crops contribute more than 85–90% of worlds caloric intake), but it is clear that in many parts of the world the use of wild plants is not negligible [1-7]. Diet surveys tend to ignore wild plants in comparison to cultivated ones [8], and this is a methodological deficiency [9]. The need for conservation of genetic resources, mostly those of wild relatives of crop plants, which can be useful in case of genetic erosion or for crop improvement, is the driving force behind our interest of studying the wild food plants [10,11]. Changing social values, depopulation of rural areas has led to erosion of traditional knowledge [12,13]. Many publications [10,11,14-23,28] have emphasised on the diversity and value of traditional vegetables. The nutritional value of traditional leafy vegetables is higher [18,24-26] than several known common vegetables. Most of these traditional leafy vegetables have a potential for income generation but fail to compete with exotic vegetables at present due to lack of awareness [[12,15] and [27]]. Consumption of traditional diets known to these societies are said to have many beneficial effects such as prevention of some age related degenerative diseases – arteriosclerosis, stroke, etc. [13]. Despite these advantages, most traditional plant foods are generally uncultivated and underutilized [5,22].

**Figure 1:**
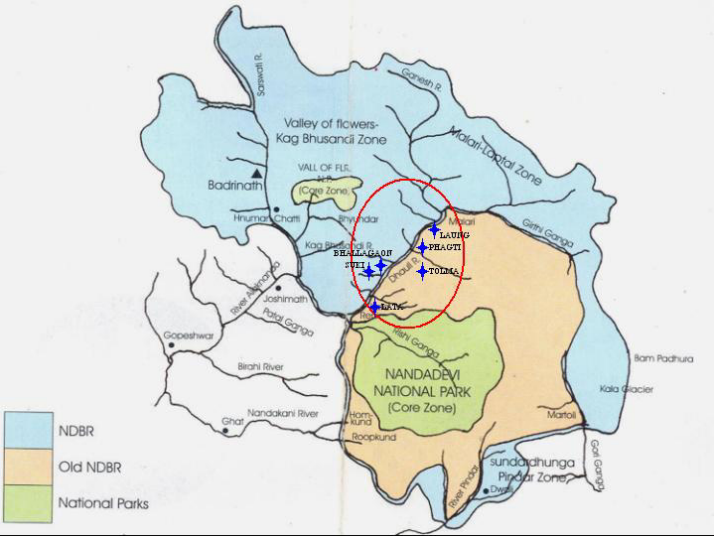
Nanda Devi Biosphere Reserve and study locations.

Uttarakhand (20° 26' and 31° 38' N latitude and 77° 49' and 80° 6' E longitude), a province (state) in India, covering an area of 53,483 km^2 ^and with the population density of 159 persons/m^2 ^is rich in diversity of wild edibles [14,17]. Nanda Devi Biosphere Reserve (NDBR), a world heritage site, occupies a special place in the biosphere reserve system of higher Himalayan region of India (Figure 1). Tolchha and Marchha sub communities of Bhotiya tribe are the main inhabitants of Niti and Mana Valleys which form buffer zone of NDBR. Besides, these traditional communities and non-tribal Khasa group also inhabited these two valleys. There are 419 households with 2253 individuals during 2001 census [29]. The sex ratio of the population is 919 and the total literacy is 36.7% [14]. Based on the distance from core zone the area could be broadly segregated into three main elevation zones i.e., Higher (2800–3600 m), middle (2400–2800 m) and lower (1900–2400 m) (Table 1). The four villages (Malari, Dronagiri, Garpak and Niti) representing higher elevation zone has maximum arable land where only one crop is grown in a year and the people practiced traditional transhumance. Middle elevation zone is represented by five villages (Tolma, Suki, Bhallagaon, Phagti and Laung) with least arable land, 3 crops in 2 year cropping cycle and the households practice short migration (modified transhumance). The lower zone is represented by three villages (Lata, Reni and Peng) where 3 crops in 2 years are cultivated and practice permanent settled agriculture on rainfed terraces. The number of cultivated crops decreased with increase in elevation. Predominant land use in these valleys is forests and alpine grazing lands. All inhabitants practice permanent cultivation of terraced slopes. Total cultivated area is 273 ha and about 8150 domestic animals are recorded in these villages. In addition to agriculture, about 11% of households have one or two members of the household involved in business and about 31% of households have one or two members in government or semi-government employment.

**Table 1:** Characteristic features of the buffer zone villages situated along an elevational gradient in Nanda Devi Biosphere Reserve, India.

Parameter	Lower	Middle	Higher
Altitude (m)	1900–2400	2400–2800	2800–3600
Transhumance	Not practiced	Practiced (short migration)	Practiced
Cropping pattern	3 crops per 2 years	3 crops per 2 years	1 crop per year
Distance from NDBR core zone (km)	5–8	3–4	<1.2
Main occupation	Agriculture	Agriculture	Agriculture
Subsidiary occupation	Animal Husbandry	Animal Husbandry	Animal Husbandry
Horticultural trees	Present	Present	Present
Number of cultivated agricultural crops	14	12	10
Number of cultivated medicinal plant species	3	4	4
Land under traditional crops (ha)	105	61	107
Land under medicinal plants (ha)	2.12	3.49	5.79
Total arable land (ha)	107.12	64.49	112.79
Name of the villages	Lata, Reni and Peng	Tolma, Suki, Bhallagaon, Phagti and Laung	Malari, Dronagiri Garpak and Niti

The climatic year consists of three distinct seasons – summer (April-June), rainy (June-September) and winter (October-February). Average annual rainfall is 928.81 mm and about 47% of this occurred during July and August itself. The mean monthly maximum and minimum temperatures varied between 24–14°C and 7.5–3°C, respectively [16]. Parent material is crystalline rocks, includes garnetiferous mica schists, garnet mica quartz schists and mica quartzite. The soils in general are deep in agricultural land, black in colour, loam to sandy loam and well to excessively drained.

The Nanda Devi Biosphere Reserve is one of the most biologically diverse areas of the western Himalaya. Though, the area lies in high altitude region, the high degree of variation in elevation resulted in diversity of microhabitats with a number of unique vegetation types. This unique feature acted both as a bridge, facilitating the influx of many taxa, and as a barrier, promoting endemism in the area. The reserve is a repository of a large variety of medicinal plants and animals having economic value. However, due to over exploitation, populations of a number of plant and animal species have become low in their natural habitats [30,31]. These species have now become rare, endangered and threatened. Some of the important taxa that are listed as threatened are *Aconitum heterophyllum *Wall ex. Royle, *Podophyllum hexandrum *Royle, *Dactylorrhiza hatagirea *D.Don, *Nardostachys grandiflora *DC. and *Taxus buccata *L. Similarly, several faunal elements such as snow leopard, Himalayan brown bear, musk deer, monal pheasant, Himalayan snowcock and snowpatridge are also said to be endangered.

The core diet of the inhabitants of the region is rice, wheat, pulses and a wide variety of local wild and semi-domesticated plants. Protein requirements are supplemented with animal products such as milk and meat. Most households grow domesticated vegetables in their kitchen gardens. Wild foods are considered by the local inhabitants in the region as necessity rather than as a supplement and are eaten frequently [14]. While several studies were conducted to document the diversity of resources [30,31] and their ethnobotanical uses [16,17,32], very few studies prioritized the species of local importance and quantified their availability, use pressure and method of use [11]. The purpose of the present study was to document plant species consumed as traditional and leafy vegetables and their ecological biodiversity in a world heritage site Nanda Devi Biosphere Reserve, India. In this study we attempted to prioritize the leafy vegetables extracted from wild and documented their status and consumption.

## Methods

Major ethnobotanical inventory of the area was conducted during 1995–2002 [16,33-35]. During these surveys data on household composition based on the village census, the diversity of wild food plants available for use was documented. Informal discussion and village walks with key informants, both adult and children were held to enhance understanding and gather information about different species of wild food plants available around the villages and in cultivated areas. Between 2002–2004 the authors surveyed six villages consisting 230 households with 1419 individuals (Table 2) to document the knowledge, frequency of use and availability status of wild leafy vegetables in the village commons and cultivated areas. About 59% households of six villages surveyed i.e. 123 households (14 of these are practicing transhumance and thus stay only for about 5 months in the valley and 109 permanent settled households) were visited by the research team during this survey. A schedule (Annexure 1) was used to collect information on personal data, traditional knowledge and priority rank for each species listed by the household. Adult female member from the household, who is responsible for food preparation, was considered as the respondent with additional information from children and adults (those assisting in collection and processing of wild leafy vegetables). Field visits were made with the informants for collection of specimens. Identification of the collected specimens was made with the help of flora of Chamoli [36] and a botanical work of the Nanda Devi National Park [30]. Herbarium specimens of the Department of Botany, H.N.B. Garhwal University, Srinagar (Garhwal) were also consulted. Prioritization of the leafy vegetables was done using a set of criteria considered to be main drivers of consumption and their availability in wild. These criteria are: (i) palatability (good, medium and low), (ii) medicinal use (yes and no), (iii) frequency of occurrence in the natural habitats (rare, intermediate and frequent), (iv) quantum of extraction (large, medium and small) and (v) existence of market value (yes and no). Based on the responses given by the 123 households surveyed, nine species either reported to be rare in occurrence or large in quantum of extraction were shortlisted. Following species satisfied these criteria viz. "doom" (*Allium semnovii *Regel), "bethua" (*Chenopodium foliolosum *Hook.), "lingra" (*Diplazium esculentum *(Retz.) Sw.), "dhol kanali" or "pachu" (*Girardinia diversifolia *(Link.) Friis.), "barmau" (*Megacarpaea polyandra *Benth.), "chandra" (*Paeonia emodi *Wall ex. Royle.), "jagra" (*Phytolacca acinosa *Roxb.), "payoom" (*Rumex nepalensis *Spreng.) and "puyanu" (*Smilacina purpurea *Wallich). "Jangli chaulai" (*Amaranthus bilatum *L.) was also prioritized for the study as we were surprised to see large extraction pressure on this genus occurring in wild though most families also cultivated the domesticated amaranth. Five families in each village were selected in January 2004 for monitoring the number of visits to collecting places, quantity of resource extracted, details of use and mode of consumption. Selection of these households involved some amount of bias as households with females having basic literacy and ability to fill in the details in data sheets was used as a criteria. This was required as the research team could not be present in all the villages at all times. However, during the visits to each of these villages, the research team personally accompanied the informant to the fields to document the extraction, processing and preparation. Using the information supplied by the informants the amount of wild leafy vegetables harvested by each family is estimated. The mean value is used to derive the total of the six villages.

**Table 2:** General profile of study villages during year 2004 in Nanda Devi Biosphere Reserve, India.

Village	Total number of households	Total population	Average family size	Number of literate persons	Average livestock/family	Total agricultural area (ha)	Average land holding size (ha)	Number of households sampled	Number of migrating families
Tolma	26	135	5.2	101	5.7	46.18	1.77	20	2
Bhallagaon	40	302	7.5	246	5.3	31.23	0.78	22	4
Suki	42	322	7.7	259	5.8	41.20	0.98	24	0
Phagti	28	141	5.0	81	6.1	42.78	1.52	17	3
Lata	75	412	5.1	302	4.4	51.23	0.68	28	5
Laung	19	107	5.6	72	6.2	16.31	0.85	12	0

Total	230	1419	6.0	1061	5.5	39.15	1.09	123	14

## Results

Twenty-one wild plant species belonging to 14 genera and 11 families are identified as being used as leafy vegetables by the informants from 123 households surveyed (Table 3). Only one plant belongs to Pteridophyta and all others to Magnoliaphyta. Two genera and five species belong to monocots (Liliopsida) and remaining are dicots (Magnoliopsida). The period of collection started from February and continued till the end of October. For most species it is short (30–60 days) but for some species such as *Chenopodium foliolosum, Phytolacca acinosa *and *Allium *spp. it is 120 days and for *Rumex *spp. it is 240 days. While palatability of most species is medium or low, for *Allium *spp., *Amaranthus bilatum*, *Chenopodium foliolosum *and *Megacarpaea polyandra *it is good and more frequently used as leafy vegetable. Except *Amaranthus bilatum*, *Chenopodium foliolosum *and *Diplazium esculentum *all others had medicinal uses also. Dried fronds of *Diplazium esculentum *are reported to be preferred animal bedding material during winters. Several of these species have market value, but only *Diplazium esculentum *and *Megacarpaea polyandra *are actually sold or purchased as fresh vegetables in the study villages. All other species with market value are sold or purchased as medicines in dried or processed forms both in the study area and exported outside. Among all the leafy vegetables only *Fagopyrum debotrys *is reported to be rare and mostly occurring in alpine pastures. Though it is reported to be rare we did not have opportunity to quantify its extraction as our sampling was restricted to settled villages only.

**Table 3:** Wild leafy vegetables and characteristics used for prioritization.

Name	Family	Collection period	Palatability	Medicinal uses	Frequency of occurring	Quantity used	Market value
*Allium humile *Kunth.	Alliaceae	March – June	Good	Yes	Frequent	Large	Yes
*Allium semnovii Regel.	Alliaceae	March – June	Good	Yes	Rare	Small	Yes
*Allium stracheyi *Baker.	Alliaceae	March – June	Good	Yes	Frequent	Large	Yes
*Allium wallichii *Kunth.	Alliaceae	March – June	Good	Yes	Intermediate	Large	Yes
*Amaranthus bilatum L.	Amaranthaceae	June – July	Good	No	Frequent	Large	No
*Chenopodium foliolosum Hook.	Chenopodiaceae	March – June	Good	No	Intermediate	Medium	No
*Diplazium esculentum (Retz.) Sw.	Dryopteridaceae	April – May	Medium	No	Frequent	Large	Yes
*Fagopyrum debotrys *D.Don	Polygonaceae	March – June	Medium	Yes	Rare	Small	No
*Fagopyrum esculentum *Moench.	Polygonaceae	March – June	Medium	Yes	Frequent	Medium	No
*Girardinia diversifolia (Link.) Friis.	Urticaceae	February – May	Low	Yes	Intermediate	Small	No
*Megacarpaea polyandra Benth.	Brassicaceae	March – April	Good	Yes	Rare	Large	Yes
*Paeonia emodi Wall. ex. Royle.	Paeoniaceae	March	Medium	Yes	Rare	Medium	Yes
*Phytolacca acinosa Roxb.	Phytolaccaceae	March – June	Low	Yes	Intermediate	Medium	Yes
*Polygonatum cirrifolium *Wall.	Convallariaceae	March – June	Medium	Yes	Intermediate	Medium	No
*Polygonatum verticillatum *L.	Convallariaceae	March – June	Medium	Yes	Frequest	Medium	No
*Rheum australe *L.	Polygonaceae	March – May	Low	Yes	Frequent	Small	Yes
*Rheum webbianum *Royle.	Polygonaceae	March – May	Low	Yes	Intermediate	Small	Yes
*Rumex hastatus *D.Don	Polygonaceae	March – October	Low	Yes	Frequent	Medium	No
*Rumex nepalensis Spreng.	Polygonaceae	March – October	Low	Yes	Intermediate	Large	Yes
*Smilacina purpurea Wallich.	Convallariaceae	March – May	Medium	Yes	Rare	Medium	Yes
*Urtica hyperborea *Jacq. ex. Wedd.	Urticaceae	February – June	Low	Yes	Frequent	Small	No

* species prioritized for detailed assessment

*Megacarpaea polyandra*, *Paeonia emodi *and *Smilacina purpurea *are processed for storing to be used as vegetable during periods of non-availability in addition to their consumption as fresh vegetable (Table 4). None of the respondents reported that a visit was made exclusively for collection of leafy vegetable from the wild. It is common for the households to collect these leafy vegetables during their visits to various places such as grazing lands, forest, crop fields and watercourses for grazing the animals, collecting fuel or fodder, tending the crop fields or collecting water etc. None of these leafy vegetables required any special processing for cooking or consumption, though removal of stings from nettle and hairs from fronds of vegetable fern could be specific requirements. All the leafy vegetables are prepared like spinach and eaten as a form of stew or cooked in oil (mainly mustard oil which is the preferred cooking medium in the study area) with salt and spices.

**Table 4:** Traditional knowledge of prioritized leafy vegetables

Name	Distribution	Consuming
Doom *Allium semnovii *Regal.	Commonly occurs in moist alpine areas. While other Alliums are domesticated, this species is still collected from wild. Low in distribution.	While fresh leaves and bulbs are used along with potato for preparation of curry, dried leaf is used as medicine and condiment.
Jungli chaulai *Amaranthus bilatum *L	Commonly occurs in wild in addition to some domesticated plants which escaped to wild.	Leaves are boiled or cut leaves are fried in cooking oil with spices.
Bethua *Chenopodium foliolosum *Hook	Commonly occurs in wild in addition to some domesticated cultivars grown in kitchen gardens	Leaves are boiled or cut leaves are fried in cooking oil with spices.
Lingra *Diplazium esculentum *(Retz.) Sw.	Frequently occurs near most areas in open forest gaps and cultivated areas	Fresh immature fronds are wiped with a cloth to remove red petiolar hairs and boiled. Boiled fronds are cut and fried in cooking oil with spices such as seeds of *Cleome viscosa *L.
Dhol kanali *Girardinia diversifolia *(Link.) Friis.	Commonly occurs near to solid wastes and agricultural wastes and on crop field margins along with *Princepia utilis *L.	Fresh leaves are boiled and mashed to remove the stings. Mashed leaves are fried in cooking oil with spices. Occasionally mashed leaves are mixed with chickpea flour, balls prepared out of this mixture is fried in cooking oil and consumed as snack.
Barmau *Megacarpaea polyandra *Benth.	Generally grows under the canopy of *Betula utilis *L and *Abies pindrow *L trees in forests. Due to excessive collection pressure becoming rare in nature, but some villagers have started cultivation in kitchen garden.	Fresh leaves are boiled or fried in cooking oil with spices. Leaves are smoked by hanging them above cooking stoves and then stored for consumption during winters.
Chandra *Paeonia emodi *Wall. ex. Royle.	Generally grows in alpine grazing lands and forests near moist areas where *Juglans regia *L or *Populas deltoides *L is dominating.	Fresh leaves are boiled with spices. Cooked leaves are fermented and preserved as a leaf cake for lean period consumption.
Jagra *Phytolacca acinosa *Roxb.	Commonly grows near forest margins and on agricultural terrace raisers.	Fresh young leaves are collected and used only during March as mature leaves are said to have poisonous substances. Fresh leaves are boiled, mashed and fried in cooking oil with spices.
Payoom *Rumex nepalensis *Spreng.	Commonly grows near to water sources.	Fresh young leaves are boiled or fried in cooking oil with spices.
Puyanu *Smilacina purpurea *Wallich.	Commonly grows under *Betula utilis *L and *Abies pindrow *L forests.	Fresh leaves are boiled or fried in cooking oil with spices. For lean periods, leaves are air dried and the smoked by keeping the dried leaves in an earthen pot hanging above cooking area.

The estimated quantity of extraction during year 2004 is maximum (2303 kg) for *Allium semnovii *and minimum (65 kg) for *Chenopodium foliolosum *(Table 5). Other important plants which reported to be extracted in large quantities are *Megacarpaea polyandra *(2125 kg) and *Paeonia emodi *(1025 kg). The estimated monetary value of the prioritized leafy vegetables shows that *Amaranthus bilatum*, *Chenopodium foliolosum *and *Girardinia diversifolia *do not have any market value. However, the seed of domesticated Amaranth have great market demand and cropping of this plant is expanding in the region. The number of households collecting a specific leafy vegetable is highest (90%) for *Megacarpaea polyandra *followed by *Amaranthus bilatum *(85%) and *Allium semnovii *(65%), *Diplazium esculentum *(65%). *Girardinia diversifolia *is used by least number (25%) of the households. This species is also reported to be used for preparation of a snack in addition to being used as vegetable. *Phytolacca acinosa *is reported to be used by 45% households although its use is restricted to less than 30 days due to harmful constituents in old leaves. The number of households consuming the wild leafy vegetables is greater than the number of households reporting to collect them except for *Diplazium esculentum *and *Phytolacca acinosa*.

**Table 5:** Estimated quantity and monetary value of wild leafy vegetables extracted in the study villages during 2004.

Name	Quantity (kg)	Monetary value (Rs)	% households collecting*	% households consuming*
*Allium semnovii *Regal.	2303	103,635	65	100
*Amaranthus *spp.	436	??	85	100
*Chenopodium foliolosum *Hook	65	??	40	100
*Diplazium esculentum *(Retz.) Sw.	178	3,916	65	65
*Girardinia diversifolia *(Link.) Friis.	89	??	25	35
*Megacarpaea polyandra *Benth.	2125	38,250	90	100
*Paeonia emodi *Wall. ex. Royle.	1025	22,550	56	65
*Phytolacca acinosa *Roxb.	236	5,192	45	45
*Rumex nepalensis *Spreng.	295	5,900	40	55
*Smilacina purpurea *Wallich.	708	17,700	48	57

US $ = Rs. 42 in 2004; ?? not sold in the markets in the villages.

* n = 123

## Discussion

The benefits of wild resources to inaccessible rural villages in Himalaya cannot be ignored. The positive relationship between the resources i.e., crops, non timber forest products and livestock indicate their concurrent relevance to livelihoods [37,38]. The number of wild leafy vegetables recorded in the present study area indicates its diversity is less as compared to other areas [26,39]. According to several informants wild green leafy vegetables increase the amount of blood in the body which is likely to refer to the high iron content of many wild greens. However, chemical analyses were beyond the scope of this study, and therefore, the information on the nutrient contents is entirely based on literature. The majority of wild edible herbs eaten typically contain high levels of important nutrients especially for diets usually high in starch [18,22,36-45]. The informants who reported these uses know perfectly well that these plants can be noxious, but they only ate some very particular parts of the plant or they use them in very small quantities. The case of *Phytolacca acinosa *in the study area elucidates the knowledge of toxicity. Such knowledge is common in other areas too [26,39,46,47]. Whilst the herbs are eaten as leafy vegetables, the majority does play an opportunistic or overlapping role as medicinals [14,16], and hence adding extra value, and thereby making them very attractive and important to the users. Wild leafy vegetables prepared as curry are eaten with bread made of ground wheat/barley/finger millet. Wild edible herbs provide important leafy vegetables for many rural households [14]. Households with limited access to cultivated vegetables such as the present region had to store dried herbs for use during the lean periods. Households that consumed herbs daily were by far the majority. This emphasizes the role of herbs in the diets of people, similarly reported in other studies [9,11,18,23,26].

There were no differences between the numbers of households that harvested wild edible herbs from microenvironments within and around the village, indicating the importance of the full range of environments. However, whilst herbs are collected from a range of environments, the amounts collected from village commons tended to be higher than that collected from distant alpine rangelands. Therefore, households do recognize all these units of land as important sources of the species they harvest and may contribute cultural significance to any management options designed for these lands. The information collected in the present study did not show any association between the income or social status and use of wild edible herbs. Households with financial means to purchase cultivated alternatives also reported that they consumed the wild edible herbs. This demonstrates the strong cultural underpinnings of the use of wild edible herbs [48] although the remoteness of the village from markets that supply cultivated species must have a role to play [49]. We did not notice any differences in preferences for wild edible herbs between households in a village. Though our data collection methods do not permit us to do any analysis, our observations during data collection clearly indicated that the knowledge about the plants is more common with older people (>35 years) as compared to young adults (13–25 years). Some of the respondents even commented that the young adults are not participating in collection and processing of these wild leafy vegetables and thus the knowledge about some of the species may disappear. This was also reported by other workers [9,39] from elsewhere. We recognize the need for collecting, preserving and documenting this knowledge as an urgent and fundamental necessity not only for maintaining the local cultural traditions but also to facilitate the research on new food sources elsewhere as well.

About two-thirds of households that consumed wild edible herbs indicated that there were sufficient herbs available for harvesting in the year of the study. However, when residents were asked to compare the current availability with the past decade, the majority was in agreement that the amount of wild herbs has decreased. This is not surprising because several households cultivated some of the wild herbs now [14]. The marginal lands and traditional agroecosystems are important sources of wild leafy vegetables as observed in this study and can make important contributions to biodiversity conservation and food security. Wild edible herbs in traditional communal areas of NDBR often grow on lands that extend from the immediate neighborhood of the built area of human settlement to the arable lands and grazing areas and at times in disturbed areas. There is also great economic potential of some species upon analyses of their nutritional and chemical composition based on species popularity and importance [28]. The high degree of coincidence of food and medicinal uses of most of these plans is particularly remarkable. In the frame of an increasing interest on pharmafoods or nutraceuticals [8,12], the study of non-crop food plants may be useful in the development of such products.

## Conclusion

Traditional knowledge is a tacit or an implicit type of knowledge that people know and apply but do not normally express. Moreover, it is localized because it gains a particular place only through experience and practice by a particular community environment. It is also dynamic and changes in the context. The way this knowledge is transmitted in not institutionalized and thus is not taught through conventional education systems. This means that traditional knowledge requires participation of individuals for learning and thus is vulnerable to loss in the face of environmental and societal changes in the rural domains. The monetization of wild edible herbs, which are mostly without formal markets, is key to understanding their value to locally unrecognized economies and hence the networks of strategies used mostly by households in remote areas for their livelihoods [50]. The study shows that wide ranges of uncultivated species are used by the majority of households as leafy vegetables. These herbs are harvested and used directly (i.e., direct-use value), by the households and often without any form of trade especially because the majority of the households often engage in the harvesting within their local environments. The direct-use value therefore represents a reasonable replacement cost for the cultivated alternatives, and offers extra cash savings for the household. Value addition through storage and commercialization could probably widen the livelihood base and thus draw attention of planners. Such strategies have been effectively used to combat vitamin and micro-nutrient deficiencies in Africa [51], and thus should be replicated in all other regions.

## Competing interests

The authors declare that they have no competing interests.

## Authors' contributions

KSR and KGS conceptualized and designed the study, SM, RKM and CPK collected field data with the participation of KSR and KGS. All authors shared the data analysis responsibility and drafted the manuscript and took part in approving the final manuscript.
